# The influencing factors of individual interest in physical education based on decision tree model: A cross-sectional study

**DOI:** 10.3389/fpsyg.2022.1015441

**Published:** 2022-10-10

**Authors:** Jia Bin Lin, Shan Shan Zhu

**Affiliations:** ^1^School of Physical Education, Changchun Normal University, Changchun, China; ^2^School of Physical Education, Northeast Normal University, Changchun, Jilin Province, China

**Keywords:** adolescents, individual interest, influencing factors, decision tree, physical education

## Abstract

To identify the key influencing factors and analyze the internal relationship among the factors of individual interest in PE, we conducted a cross-sectional survey of a large sample of Chinese young students based on the decision tree model. A total of 3,640 young students (*M*_age_ = 14.16; 7–18 years; SD = 2.66, 47% boys) were investigated by using six questionnaires, including individual interest in physical PE, self-efficacy, achievement goals, expectancy value in PE, PE knowledge and skills and PE learning environment. Results showed there were a total of seven variables entered into the decision tree model, which was 3 layers high, including 38 nodes. The root node was expectancy value which was divided by sports knowledge and skills and self-efficacy. The third layer included mastery-approach goal, family sports environment, performance-avoidance goal and gender. The results depict that expectancy value of PE was the most important influencing factors of adolescent students’ individual interest in PE in this study, and the other important factors were sports knowledge and skills, self-efficacy, mastery-approach goal, family sports environment, performance-avoidance goal, and gender, respectively. The implications for PE are: (1) Improve the status of the PE curriculum and enhance students’ recognition of the value of PE; (2) Strengthen the teaching of knowledge and skills to avoid low-level repetitive teaching; (3) Enhance success experience and foster sports self-efficacy; and (4) Establish reasonable sports goals to foster individual interest in sports learning.

## Introduction

To actively engage with and persist on a learning task, students need to be sufficiently motivated (Rotgans and Schmidt, 2017). Renninger and Hidi (2016) regarded interest as a powerful motivator variable that directs students’ attention to specific objects and stimuli and guides their engagement towards specific activities. In educational research, researchers conceptualized interest as situational and individual (Chen and Darst, 2002). Situational interest is a relatively transient reaction to highly stimulating factors in the immediate environment, whereas individual interest is a relatively long-term preference for a particular subject or activity (Palmer et al., 2017).

In physical education (PE), systematic research on interest has mainly investigated SI and evidence has been accumulated regarding its sources, motivational function, and relationship with learning (Chen and Wang, 2017). Researchers concluded that individual interest has an important effect on performance and cognitive functioning, as students who are interested in a domain or task have been shown to pay more attention, persist for longer periods of time, and acquire more and qualitatively different knowledge than individuals without such an interest (Hidi, 1990; Palmer et al., 2017). Despite the role of individual interest has been general accepted, it has been subjected to limited empirical testing in educational settings (Chen and Wang, 2017).

Given that the most recent global estimates show that more than three-quarters (81%) of adolescents do not meet the recommendations for aerobic exercise, as outlined in the 2010 Global Recommendations on Physical Activity for Health (Bull et al., 2020). More than about 60% of children and adolescents do not meet the recommended amount (Zhu, 2021). In the face of the problem of declining physical health and insufficient participation in physical activity among teenagers around the world, it is particularly important to deeply explore the influencing factors of individual interest. However, the influencing factors of individual interest in PE have rarely been studied. Consequently, it is not clear that to what extent each influencing factor will facilitate or hinder the development of individual interest in PE. And there is a lack of targeted strategies on how to improve students’ individual interest in PE in different situations, which makes it difficult to explain and improve the reality of low individual interest in PE among young people in China.

Research showed that the decision tree model analysis method could not only obtain a more intuitive relationship diagram between various influencing factors, but also identify the most critical influencing factors of individual interest in PE, construct a clearer classification standard, and dig deeper into the role of each factor (Henrard et al., 2015; Zhao et al., 2020). This method has an in-depth theoretical basis and more targeted practical guidance significance for cultivating individual interest in youth sports participation. Therefore, in view of the practical background of low individual interest in youth sports and lack of targeted promotion strategies, this study adopted decision tree model analysis methods to analyze 3,640 people based on comprehensive consideration of ethical issues (voluntary rights, the right to know, and privacy protection, etc.). A survey of young students aged 7–18 was carried out to reveal the key factors influencing young students’ individual interest in PE and the relationship among the factors, and to further explore the implications of the research results on the promotion of individual interest in young students, aiming to provide a useful reference for improving youth sports learning individual interest.

### Social cognitive theory and individual interest

Bandura (1986) first proposed the social cognitive theory, then the theory was widely applied and carried out a large number of empirical studies. Social cognitive theory is widely used by researchers to analyze the influencing factors of individual behavior (Sumak et al., 2011; Zhou et al., 2020). This theory analyzes the influencing factors of individual behavior in detail and holds that the generation or change of individual behavior is not only affected by external environmental factors, but also influenced by their own internal psychological factors (Bandura, 1986; Li and Hua, 2022). The social cognitive theory explains human behavior using a three-way model in which environment, personal factors, and behavior interact continuously (Shamizadeh et al., 2019; Sebastian et al., 2021), and emphasize the role of self-efficacy, expectancy-value, achievement goals, knowledge and other factors.

### The relationship between environment factors and interest

Traditional behavioral theory points out that individual behavior depends entirely on external environmental stimuli, despite being greatly questioned and criticized, the role of the environment cannot be ignored. From the perspective of space, environmental factors include three aspects: family environment, school environment, and social environment. Results showed that parents impacted the trajectory of participants’ athletic careers and their general approach toward sport (Erickson et al., 2017). The local environment can affect an individual’s interest, and the space available for sports, the distance to facilities, and quality of the equipment all naturally impact willingness to participate (Gomes et al., 2016). Participants who lived in rural settings were less interested in recreational sports than their urban counterparts (Chen et al., 2017). Existing evidence suggests that the influence of environmental factors on interest is mediated or modulated by other variables, such as self-efficacy (Halim et al., 2021), and the action effect of environmental factors still needs further research.

### The relationship between expectancy-value and interest

Motivated behavior is characterized by voluntary choices, persistent effort, and achievement, which are directly associated with students’ expectancy for success and perceived value in specific activities (Chen et al., 2008). The expectancy-value theory argued that students’ expectancy-value motivation directly predicts their achievement and behavior choices, and that student achievement over time predicts their behavior choices (Eccles et al., 1983; Eccles and Wigfield, 1995). Expectancy belief and task values have been identified as predictors for both physical activity participation intention (Xiang et al., 2003) and successful performances in physical education (Gao et al., 2009). Findings in other areas have shown that task value (Bai et al., 2020) and utility value (Hulleman et al., 2010; Akcaoglu et al., 2018) and interest are closely related.

### The relationship between self-efficacy and interest

Self-efficacy is a positively focused ability belief that describes a person’s perception of his ability to successfully complete a specific task (Bandura, 1977). It was found to be as important as value in educational settings and was an important predictor of achievement (Fryer and Ainley, 2019; Nuutila et al., 2021). While the majority of self-efficacy research focused on task-level outcomes, Bandura (2011) has clarified that self-efficacy are also related to long-term pursuits such as skill development have developed over time and are not limited to individual events. Increasing empirical evidence supports the important role of self-efficacy in benefits, with long-standing theories suggesting that the two are interconnected over time (Fryer and Ainley, 2019; Nuutila et al., 2020).

### The relationship between achievement goals and individual interest

Researchers have identified two types of achievement goals that students adopt: mastery and performance goals (Nicholls, 1984; Dweck, 1986). Further studies subdivided these achievement goals into approach and avoidance components, presented four categories: mastery-approach goal, performance-approach goal, mastery-avoidance goal, and performance-avoidance goal (Elliot and McGregor, 2001). Numerous studies found a positive correlation between mastery-approach goal and individual interest, but the relationship between performance-approach goals and individual interest is still unclear (Hulleman et al., 2010; Linnenbrink-Garcia et al., 2013). Roure et al. (2021) found that the positive correlations between both mastery-approach and performance-approach and individual interest, and confirmed the key role played by students’ mastery-approach goal when considering its relationship with students’ individual interest (Roure and Lentillon-Kaestner, 2021). The meta-analysis results show that, relative to performance-approach and performance-avoidance goals and no-goals, induced mastery-approach goals enhanced performance (Huang, 2011, 2012), but not motivation (Noordzij et al., 2021). Overall, more research is needed to clearly understand the relationship between students’ achievement goals and their individual interest.

### The relationship between knowledge, skills and interest

Reviews have consistently pointed that prior knowledge is one of the most important individual difference brought to the learning experience (Lin and Chai, 2019; Fryer et al., 2021). Prior knowledge can account for 30–60% of the variance in future learning (Tobias, 1994). Knowledge refers to one’s understanding of a given domain in either a declarative (factual) or procedural (skillful execution) form (Alexander et al., 1991). A majority of studies showed that the relationship between interest and knowledge may be two-way, students with high individual interest in a field are likely to continue to acquire additional knowledge in that field as they are naturally drawn to the subject and are willing to spend more time and effort to learn more about the subject (Tobias, 1994). And in return, increased knowledge is likely to strengthen the interest, because the expanded knowledge affords the individual to extend the knowledge base on which interest is developed and sustained. Prior knowledge determines interest in learning in physical education (Zhang et al., 2016), interest is a by-product of knowledge (Rotgans and Schmidt, 2017).

## The present study

Based on previous research, the purpose of this study was to explore the influencing factors of individual interest in PE from three aspects: demographic factors, environmental factors, and individual factors. Variables investigated include gender, school location, sports environment, expectancy value, sports knowledge and skills, self-efficacy, and achievement goals. As Henrard et al. (2015) argued, the decision tree model was an important classification technique in data mining, and optimal segmentation for multiple types of variables was an important function of this method. Therefore, this study chose the decision tree model as the main method to analyze the importance and internal relationship of each influencing factor. These analyses have theoretical implications for how individual interest develops across the PE learning process, and they are of practical concern to educators seeking to enhance students’ individual interest and sports participation independently.

## Materials and methods

### Participants

The present study sample consisted of 3,640 students (*M*_age_ = 14.16; 7–18 years; SD = 2.66, 47% boys) from 110 PE classes, taken from 11 cities located in the Northeast, East, Central, and West regions of China. Students were in grades 1–12. Class sizes ranged from 20 to 65 students per class. Permission to conduct the study was granted by the ethical board of the host university, and agreement was also obtained from the principals of the participating schools.

### Materials

#### Individual interest

The Chinese Individual Interest Scale in PE (Lin, 2019) was used to measure students’ individual interest. As Rotgans (2015) argued, the instrument of individual interest should measure at least the following three key components of the definition: (a) willingness to reengage with specific content, (b) positive emotions, and (c) increased value for the topic. Take willingness to participate (e.g., ‘I often take part in sports activities in my spare time’), emotional experience (e.g., ‘Participating in sports activities brings me a lot of fun’) and value embodiment (e.g., ‘I want to work in sports or sports-related industries in the future’) as three dimensions to compile the questionnaire of individual interest in PE. Each of these three dimensions consists of three items. These nine items were randomly arranged and each was rated on a five-point Likert scale, ranging from 1 = ‘strongly disagree’ to 5 = ‘strongly agree’. Lin (2019) established the construct validity of the Chinese Individual Interest Scale in PE using exploratory and confirmatory factor analyses (*χ*^2^/*df* = normed fit index (NFI) = 0.97, comparative fit index (CFI) = 0.99, Tacker-Lewis index (TLI) = 0.98, incremental fit index (IFI) = 0.99, and root mean squared error of approximation (RMSEA) = 0.045). The internal consistency (Cronbach’s alpha) and test–retest reliability factor for willingness to participate (0.81, 0.87), emotional experience (0.86, 0.84), value embodiment (0.73, 0.82) and for the total scale (0.90, 0.85) among the grade 1–12 school students.

#### Environment factors for PE

Investigate the sports learning environment from three aspects: school sports environment (including school sports facilities, equipment, PE teachers, sports activities and sports curriculum development, etc.; e.g., ‘How is your school’s sports facilities?’), family sports environment (including family sports equipment, parents’ support, family sports atmosphere, etc.; e.g., What is the atmosphere of your family sports activities?’) and social sports environment (including social sports venues, social sports activities and clubs, etc.; e.g., How about the surrounding sports clubs and activity centers?’). The questionnaire consists of 16 randomly arranged items, and each was rated on a five-point Likert scale, ranging from 1 = ‘very bad’ to 5 = ‘very good’. The construct validity of the questionnaire was established by means of exploratory and confirmatory factor analysis (Byrne, 2001), *χ*^2^/*df* = 1.592, NFI = 0.94, CFI = 0.98, TLI = 0.97, ILI = 0.98, RMSEA = 0.048. The internal consistency (Cronbach’s alpha) and test–retest reliability factor for school sports environment (0.90, 0.92), family sports environment (0.86, 0.91), social sports environment (0.83, 0.88) and for the total scale (0.93, 0.90).

#### Expectancy-value

Students’ expectancy beliefs and task values were measured using a modified Chinese Expectancy-Value Questionnaire for PE (Eccles and Wigfield, 1995; Chai and Lin, 2019). The questionnaire is a 5-point Likert scale of 11 items. Five items were designed to measure expectancy beliefs and six items to measure attainment (importance), intrinsic (interest), and utility (usefulness) values. In completing the questionnaire, students were asked to respond to the items by indicating their preference on the five-point scale attached to each item. For example, in responding to the item “How important do you think PE is for you?” the student can choose a number between 1 and 5, with 5 indicating “very important” and 1 indicating “not important.” The descriptors “very important” and “not important” are printed explicitly on the EVQ to avoid confusion (Zhu et al., 2012). Chai and Lin (2019) confirmed its construct validity by means of confirmatory factor analysis and found that the measurement model of Chinese EVQ was well preserved with *χ*^2^/*df* = 2.73, NFI = 0.99, CFI = 0.99, TLI = 0.99, ILI = 0.99, RMSEA = 0.020. The internal consistency (Cronbach’s alpha) and test–retest reliability factor for expectancy beliefs (0.89, 0.88), attainment values (0.78, 0.89), intrinsic values (0.84, 0.91), utility values (0.84, 0.85) and for the total scale (0.80, 0.87).

#### Self-efficacy

The Generalized Self-Efficacy Scale (GSES; Schwarzer and Jerusalem, 1995) was used to measure students’ self-efficacy. The questionnaire consists of 10 randomly arranged items, and each was rated on a five-point Likert scale, ranging from 1 = ‘strongly disagree’ to 5 = ‘strongly agree’. The internal consistency (Cronbach’s alpha) and test–retest reliability factors in this investigation were 0.86 and 0.89.

#### Achievement goals

The 2 × 2 Achievement Goals Questionnaire (AFQ-PE) compiled by Guan (2004) was used to measure students’ achievement goals. The scale includes four dimensions: master-approach goal, master-avoidance goal, performance-approach goal, and performance-avoidance goal. Each of these four dimensions consists of three items. These 12 items were randomly arranged and each was rated on a five-point Likert scale, ranging from 1 = ‘strongly disagree’ to 5 = ‘strongly agree’. The internal consistency (Cronbach’s alpha) and test–retest reliability factors in this investigation were 0.89 and 0.88.

#### Sports knowledge and skills

Use a self-reporting questionnaire to evaluate students’ sports knowledge and skills. The questionnaire consists of six randomly arranged items, and each was rated on a five-point Likert scale, ranging from 1 = ‘strongly disagree’ to 5 = ‘strongly agree’. The items are as follows: (1) ‘I know more about sports knowledge than most of my classmates’; (2) ‘I am familiar with many sports’; (3) ‘I am familiar with many sports’; (4) ‘I have many sports skills better than most of my classmates’; (5) ‘At least one sports skill I master better than most of my classmates’; (6) ‘I have many sports skills better than most of my classmates’. The internal consistency (Cronbach’s alpha) and test–retest reliability factors in this investigation were 0.89 and 0.91.

### Procedure

Data came from a cross-sectional study investigating 7–18 year-old teenage students’ individual interest in PE. Assessments were completed over two-month periods in spring 2019 and fall 2020. All questionnaires will be distributed, filled out, and collected by 11 graduate students who have undergone strict training immediately after the PE class. In order to ensure that all the students fully understand the meaning of the questions and options, the graduate students read the questions aloud to the first and second grade students in elementary school, making corresponding explanations. Then ask students to fill out the questionnaire and raise their hands whenever they encounter problems during the filling process. All in all, the testing of each child took about 20 min.

### Statistical analyses

The data was analyzed using SPSS for Windows Version 22.0. Because all of the data in this study were gathered *via* questionnaires and all items were completed by young students, there may be common method bias in the research supporting this thesis (Gorrell et al., 2011; Mackenzie and Podsakoff, 2012). First, the Harman single factor test method was used to conduct common method bias. The specific method was to perform Principal Component Analysis (PCA) on all questionnaires and scale items. The results showed that there are 15 factors with a characteristic value greater than 1, and the variance explained by the first factor is 25.30%, which is less than the critical standard of 40% (Cao and Chi, 2016). The results showed that there was no serious common method bias problem in this study.

Subsequently, create a decision tree model. According to the characteristics of the large sample, multiple indicators, continuous variables, and categorical variables in this study were compared to the accuracy of each model, finally determining the optimized CHAID model for decision tree analysis (Henrard et al., 2015). Among all the variables, gender, grade, and school location were category variables. The two grades of the gender variable “male” and “female” were marked as 1 and 2 respectively, the 12 grades of grade variable “1 ~ 12” were marked as “1 ~ 12” respectively, and the variables of the city and village where the school is located were marked as 1 and 2 respectively; other variables are continuous variables, and the best cut-off point is identified and split by the decision tree model. The model parameters were set as follows: the maximum depth of the decision tree is 5, the minimum number of cases of influencing factor nodes is 200, the minimum number of cases of sub-nodes is 100, the minimum change value of the Gini coefficient is 0.0001, and the recognition accuracy rate of the 10-level cross-validation model is adopted (Cao and Chi, 2016).

The rules for ranking the importance of various factors affecting individual interest in PE are: (1) sort according to the position of the node where the variable is located, the closer the variable is to the root node, the greater the impact on the target variable; (2) At the same level of branches, we compared the value of *p* and Chi-square of each variable. The smaller the value of *p*, the greater the impact on the target variable. If the value of *p* is equal, compare the chi-square value; (3) At non-terminal nodes, if the sample size of the variable is less than 10, the variable is not regarded as an important one.

## Result

### Descriptive statistics

Table 1 shows the descriptive statistics as well as the correlation matrix between the measures of the study for the whole sample across different grade students. The results show that the correlation among each variable and between each variable and individual interest have reached a significant level (*p* < 0.05).

**Table 1:** Means (and SD), and intercorrelations between environment factors, expectancy-value, self-efficacy, sports knowledge and skills achievement goals and individual interest.

Variable	*M*	SD	1	2	3	4	5	6	7	8	9	10	11
1. Individual interest	3.31	0.86	1										
2. School environment factors	4.04	0.80	0.337	1									
3. Family environment factors	3.65	0.92	0.456	0.527	1								
4. Social environment factors	3.58	1.04	0.410	0.545	0.649	1							
5. Expectancy-value	3.52	0.72	0.669	0.341	0.369	0.336	1						
6. Self-efficacy	3.40	0.79	0.549	0.294	0.407	0.365	0.416	1					
7. Sports knowledge and skills	3.23	0.92	0.652	0.248	0.423	0.372	0.528	0.539	1				
8. Master-approach goal	3.74	0.96	0.596	0.426	0.386	0.353	0.525	0.491	0.507	1			
9. Master-avoidance goal	3.46	0.98	0.353	0.231	0.253	0.209	0.267	0.335	0.376	0.533	1		
10. Performance-approach goal	3.26	1.02	0.361	0.172	0.233	0.195	0.305	0.416	0.493	0.406	0.557	1	
11. Performance-avoidance goal	3.31	1.01	0.174	0.156	0.172	0.177	0.107	0.267	0.294	0.248	0.460	0.534	1

### Construction of decision tree model

**Figure 1:**
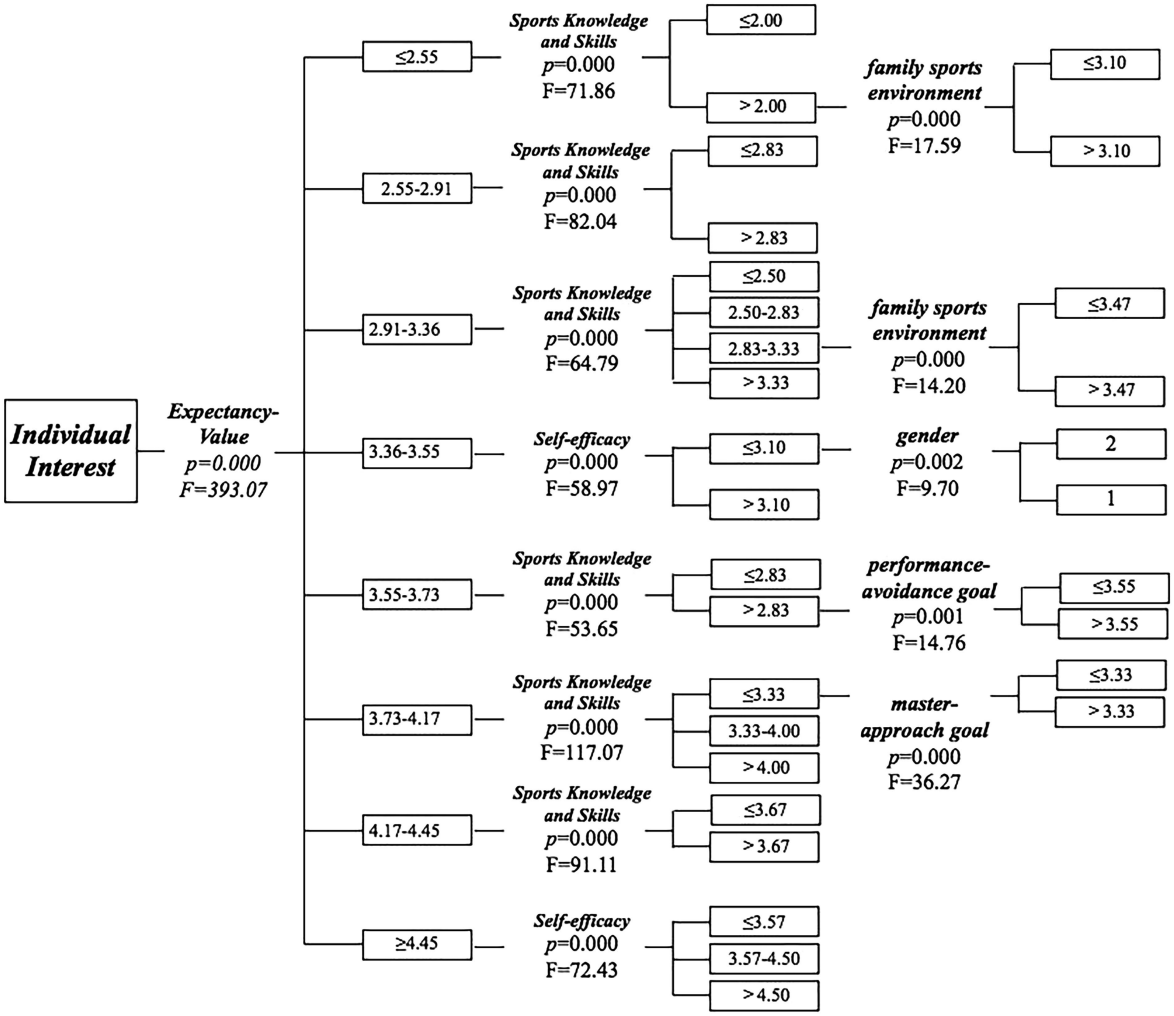
The decision tree model of the seven influencing factors, including expectancy-value, sports knowledge and skill mastery, self-efficacy, mastery-approach goal, family sports environment, performance–avoidance goal, and gender of individual interest. The asterisk indicates a statistically non-significant level of alpha. 05.

The decision tree model of the influencing factors on individual interest in PE created by this research has 3 layers and 38 leaf nodes (see Figure 1). The results showed that a total of seven variables entered the model, in order of importance. They are: (1) expectancy-value; (2) sports knowledge and skill mastery; (3) self-efficacy; (4) mastery-approach goal; (5) family sports environment; (6) performance-avoidance goal and (7) gender.

At the first layer of the decision tree structure, students’ individual interest in PE was divided into 8 nodes according to “expectancy-value,” and the difference between each node reached a significant level (*F* = 393.07; *p* < 0.05). The higher the students’ expectancy-value, the greater their individual interest in PE. Students whose expectancy-value score ≥ 4.45 had the highest individual interest, and students whose score ≤ 2.55 had the lowest individual interest.

At the second layer of the decision tree structure, 8 nodes of students’ expectancy-value in PE were divided into 20 nodes according to “sports knowledge and skills” and “self-efficacy” (see Figure 1). Students whose expectancy-value scores were ≥ 4.45 and were between 3.36 and 3.55 were divided into 3 (self-efficacy scores: < 3.57, 3.57–4.5, > 4.5; *F* = 72.43, *p* < 0.05; the node no longer grows) and 2 (self-efficacy scores: ≤ 3.10, > 3.10; *F* = 58.98, *p* < 0.05) nodes, respectively, according to their “self-efficacy.” The higher the self-efficacy, the higher the expectancy-value score. In the self-efficacy score ≤ 3.10 group, there are gender differences in the self-efficacy scores of students, and boys’ self-efficacy scores are higher than those of girls.

Students whose expectancy-value scores in the other six ranges were divided into 2 (sports knowledge and skills scores: ≤ 2.00, > 2.00, *F* = 71.86, *p* < 0.05), 2 (sports knowledge and skills scores: ≤ 2.83, > 2.83, *F* = 82.04, *p* < 0.05), 4 (sports knowledge and skills: ≤ 2.50, 2.50–2.83, 2.83–3.33, > 3.33, *F* = 64.79, *p* < 0.05), 2 (sports knowledge and skills: ≤ 2.83, > 2.83, *F* = 53.65, *p* < 0.05), 3 (sports knowledge and skills: ≤ 3.33, 3.33–4.00, > 4.00, *F* = 111.07, *p* < 0.05) and 2 (sports knowledge and skills: ≤ 3.67, > 3.67, *F* = 91.11, *p* < 0.05) nodes, respectively, according to their “sports knowledge and skills,” the higher the sports knowledge and skills score of students, the higher the expectancy-value score. At the last layer of the decision tree structure, the sports knowledge and skills were divided into 8 nodes: (1) the sports knowledge and skills scores > 2.00 group were divided into 2 nodes (≤ 3.10, > 3.10, *F* = 17.59, *p* < 0.05) according to their family sports environment; (2) the scores between 2.83 and 3.33 group were divided into 2 nodes (≤ 3.47, > 3.47, *F* = 14.20, *p* < 0.05) according to their family sports environment; (3) the scores >2.83 were divided into 2 nodes (≤ 3.55, > 3.55, *F* = 14.76, *p* < 0.05) according to their performance-avoidance goal; (4) the scores ≤ 3.33 group were divided into 2 nodes (≤ 3.33, > 3.33, *F* = 36.27, *p* < 0.05) according to their mastery-approach goal. In each group, students’ sports knowledge and skill scores increase with the increase of branch indicators.

### Decision tree model evaluation

The accuracy recognition result of the 10-layer cross-validation model shows that the accuracy of the decision tree model of the factors affecting individual interest in PE of primary and middle school students constructed in this research was 90.88% (see Table 2).

**Table 2:** Recognition accuracy rate of the model of factors affecting individual interest in PE of primary and middle school students.

	*N*	%
Accurate	3,380	90.88
Error	332	9.12
Total	3,640	100

## Discussion

The purpose of the present study was to identify the key influencing factors of individual interest in PE among primary and middle school students in China. To rank the influencing factors according to their importance accurately, we selected a total of 13 variables as the influencing factors of individual interest in PE for decision tree analysis, including gender, grade, school location, school sports environment, family sports environment, social sports environment, expectancy-value, self-efficacy, sports knowledge and skills, master-approaching goal, master-avoidance goal, performance-approach goal, and performance-avoidance goal, and conducted a large sample of 3,640 students selected from 11 cities. The selected questionnaires and scales have been tested for reliability and validity and could be used as measurement tools for this study. There was no common method bias among all the questionnaires and scales.

The decision tree adopts a top-down recursive approach to compare and evaluate the attribute values of nodes within the decision tree and determine the branch down from the node based on the different attribute values (Zhang et al., 2020). The decision tree algorithm has been widely used in different fields since its introduction (Tao et al., 2016). Not only that, the decision tree analysis could also identify the key influencing factors of individual interest in PE of primary and middle school students in China, and make up for the shortcomings in the current research on many influencing factors of sports learning interests. Decision tree algorithm models include CHAID, C5.0, QUEST, and C&R. Combining the characteristics of the large sample, multiple indicators, and the simultaneous existence of continuous variables and categorical variables in this study, the accuracy of related models is compared, and the optimized CHAID model is selected (Zhao et al., 2020). The results show that the constructed decision tree model of the factors affecting individual interest in PE of primary and middle school students was 3 layers high, divided into 38 leaf nodes, and the decision tree model was lush and leafy. In addition, the accuracy of the model was as high as 90.88%, which is satisfactory for the needs of this research.

There was a total of seven variables entered into the decision tree model in this study. In order of importance, they were: expectancy-value, sports knowledge and skills, self-efficacy, mastery-approach goal, family sports environment, performance-avoidance goal, and gender. Among them, except for the two variables of family sports environment and gender, the other variables are all individual factors, which is consistent with previous research conclusions (Chai and Lin, 2019). This result is in line with the ternary interactive determinism of social cognitive theory (Bandura, 1977), which argues that individual factors (expectancy value, self-efficacy, knowledge, and goals are important individual factors), environmental factors, and behavioral factors are dynamic interactions (Bandura, 1989, 2001; Chiu et al., 2007; Jeng et al., 2022).

Previous studies have suggested that expectancy beliefs and perceived task values, a source of situational interest, were positively related to after-school physical activity (Chen et al., 2014). In this study, expectancy value was located at the root node of the individual interest decision tree model, indicating that it was the most important factor affecting individual interest. Eccles et al. (1983) argues that students’ learning interest stems from their expectancy beliefs and the value of the task, collectively referred to as “expectancy value.” Expectancy beliefs are students’ perceptions of the possibility of success in the upcoming learning task, and task value, including achievement value, intrinsic value, utility value, and cost, is the student’s perception of the value of the learning task. Previous studies have suggested that expectancy is positively associated with interest (Xu et al., 2020), and task expectancy motivation could predict students’ future interest in math at the individual and class level (Ruiz-Alfonso et al., 2021). This also appeared to be the case in the present study. Not only that, this research further proved that expectancy value was the most important influencing factor of individual interest in PE among all the factors of social cognition theory investigated in this study. It is not difficult to find that the current reality of the implementation of the physical education curriculum in primary and secondary schools in China makes it difficult to improve the life expectancy value of students: (1) Poor attendance rate of PE courses, according to the survey conducted by the State Sports General Administration (2014), 53.9% of the fourth graders have less than three sessions of PE per week, the serious over-standards of Chinese, mathematics, physics, and other courses were in sharp contrast with this; (2) Poor PE teachers’ team. The number of full-time PE teachers is seriously insufficient, and part-time PE teachers account for a large proportion. And these teachers mostly adopt the “shepherd type,” which makes it difficult to satisfy the students’ interest in classroom sports (Mao et al., 2019); (3) Playground and ground equipment need to be further improved. All kinds of phenomena reveal that the attention paid to the PE curriculum of primary and middle school students in China is not up to standard, and still needs to be improved. Therefore, we appeal to improving students’ expectancy value of PE by enhancing the attention of PE curriculum, teachers’ literature, and teaching environment, so as to improve students’ individual interest.

The relationship between knowledge and interest has always received widespread attention. Almost all researchers take interest as an independent variable and individual interest as a dependent variable, believing that interest is the reason for acquiring knowledge (Schraw and Lehman, 2001, 2009; Tomlinson et al., 2003). Rotgans and Schmidt (2017) examined the causal relationship between students’ individual interest and knowledge acquisition using cross-lagged panel analysis; results showed that individual interest was not the cause but the consequence of the process of learning: individual interest as an affective by-product of learning. In this study, there were 6 groups of students’ expectancy values classified according to their sports knowledge and skills. According to the findings, sports knowledge and skills were the second most important influencing factor of individual PE interests. At present, the phenomenon of low-level repetitive teaching in the PE curriculum is more common in China. After years of study, students still cannot master one or two sports skills proficiently, let alone form a stable individual interest (Mao et al., 2019). Therefore, we argue that while using novel teaching activities to stimulate students’ situational interest, we should also teach students some sports knowledge and skills to cultivate their individual interest, which is obvious, but often overlooked.

At the second level of the decision tree structure, there were 2 groups of students’ expectancy values classified according to their self-efficacy. The results showed that students’ self-efficacy was the third important influencing factor of individual interest in PE. The results of previous studies show that individual interest and self-efficacy are positively correlated (Armstrong et al., 2009). This growing body of empirical evidence supporting the important role of self-efficacy within an interest is buttressed by long-standing theory suggesting that the two are reciprocally linked over time (Fryer et al., 2016, 2019; Nuutila et al., 2020, 2021). Fryer et al. (2021) used the potential curve to analyze the role of self-efficacy between knowledge development and individual interest, which lends further support to the critical role played by self-efficacy beliefs within the development not only of knowledge but also of individual interest as a learning outcome. The role of self-efficacy in determining individual interest has been confirmed by a large number of research results. The emotional experience of sports participation, especially a successful experience, is helpful to the establishment of self-efficacy. Therefore, we appeal to strive to enable each student to obtain successful experiences in the process of sports participation and cultivate their sports confidence so as to obtain a long-term and stable individual interest in PE.

There are five branches in the third layer of the decision tree model, and master-approaching goal is the most important variable in this layer, followed by the family sports environment, performance-avoidance goal, and gender. The research of Harackiewicz et al. (2000, 2008) showed that achievement goals can predict students’ interest and academic achievement in the short term or long term. Among them, mastering goals can effectively predict students’ interest, but there was no predictive effect on academic achievement; on the contrary, achievement goals can effectively predict students’ academic performance, but they cannot predict their learning interest. This research examines the influence of achievement goals on individual interest from four aspects: performance-approach goal, performance-avoidance goal, master-approach goal, and master-avoidance goal. The results showed that master-approach goal and performance-avoidance goal could predict students’ individual interest in PE, and the effort of the master-approach goal was better than the performance-avoidance goal.

In addition, family sports environment and gender have also entered the decision tree model of students’ individual interest in PE, but school sports environment, social sports environment, grade, and school location did not correspondingly. The results of this study confirmed the important role of the family sports environment in the development of students’ individual interest in PE once again. Knight et al. (2016) identified a number of individual and environmental influences on parental involvement in youth sports, and the results showed that parents were involved as supporters, coaches and managers, and providers of opportunities. Parents’ past experiences in sports and as a sport parent, their beliefs, goals, and values, the youth sport context, their concerns regarding others, and their own behavior can affect youth sports. Erickson et al. (2017) used a qualitative methodology to explore the role of significant others in this domain, and the results showed that the parent-athlete relationship influenced athletes’ lives in and beyond sport and could shape athletes’ attitudes, experiences, and behaviors toward doping. Parents are the most important part of the family sports environment for primary and middle school students, so we call on all children’s parents to pay attention to their attitudes towards sports activities and establish a positive family sports environment for their children. At the same time, we have also discovered the weak role of gender in the decision tree model of individual interest influencing factors. This is consistent with the results of previous research and is a current development trend of Chinese students’ individual interest in PE (Lin, 2019).

## Conclusions, limitations and future directions

The current study investigates the factors affecting individual interest of primary and secondary school students based on social cognitive theory and ranks multiple influencing factors in order of importance using decision tree model analysis. It has been demonstrated that the most important factor influencing individual interest in PE is expectancy value, which is followed by sports knowledge and skills, self-efficacy, mastery-approach goal, family sports environment, performance-avoidance goal, and gender. The implications for PE are: (1) improve the status of the PE curriculum and enhance students’ recognition of the value of PE; (2) strengthen the teaching of knowledge and skills to avoid low-level repetitive teaching; (3) improve success experience and cultivate sports self-efficacy; and (4) set reasonable sports goals to cultivate individual interest in sports learning.

This study adopted a large sample method to collect a total of 3,640 primary school students nationwide, but for China, with a population of 1.3 billion, the sample size was still slightly insufficient. In addition, this study did not analyze the differences in factors affecting students’ individual interests in PE according to different grades or stages of learning. Future work might further expand the sample size to make the sampling more representative, and it might also analyze the differences in students’ individual interests in stages.

The second limitation lies in the lack of data; data consisted solely of self-reported measures, and all questionnaires and scales were filled out by student groups. However, we focused on students’ subjective motivational perceptions of individual interest, and self-report was the rule rather than the exception. Nonetheless, we recognize the methodological problems that are likely to occur when relying exclusively on self-reported measures (Knogler et al., 2015). Self-reported data potentially suffers from inaccuracy, especially at earlier stages of interest development, when people may lack meta-cognitive awareness of their interest (Renninger and Su, 2012). Therefore, we encourage future work to use multiple sources of information, and to further determine the importance of influencing factors on individual interest in PE.

## Data availability statement

The raw data supporting the conclusions of this article will be made available by the authors, without undue reservation.

## Ethics statement

The studies involving human participants were reviewed and approved by Changchun Normal University work place. Written informed consent to participate in this study was provided by the participants' legal guardian/next of kin.

## Author contributions

JL: conceptualization, methodology, software, investigation, formal analysis, and writing—original draft. SZ: data curation and writing—original draft. All authors contributed to the article and approved the submitted version.

## Funding

This article is supported by the Humanities and Social Science Fund Project of the Ministry of Education of China (22YJC89057), the Humanities and Social Sciences Project Fund of the Jilin Province (2022C106 and 2021C100), the Humanities and Social Sciences Project Fund of the Jilin Provincial Department of Education (JJKH20210908SK and JJKH20180050SK), and the Humanities and Social Sciences Fund of Changchun Normal University ([2020]003).

## Conflict of interest

The authors declare that the research was conducted in the absence of any commercial or financial relationships that could be construed as a potential conflict of interest.

## Publisher’s note

All claims expressed in this article are solely those of the authors and do not necessarily represent those of their affiliated organizations, or those of the publisher, the editors and the reviewers. Any product that may be evaluated in this article, or claim that may be made by its manufacturer, is not guaranteed or endorsed by the publisher.
